# Identification of potential diagnostic biomarkers for Parkinson's disease

**DOI:** 10.1002/2211-5463.12687

**Published:** 2019-07-03

**Authors:** Fenghua Jiang, Qianqian Wu, Shuqian Sun, Guanghui Bi, Ling Guo

**Affiliations:** ^1^ Department of Neurology Dongying People's Hospital China; ^2^ Department of Rheumatology Dongying People's Hospital China

**Keywords:** biomarker, differentially expressed genes, integrated analysis, Parkinson's disease

## Abstract

The identification of biomarkers for early diagnosis of Parkinson's disease (PD) prior to the onset of symptoms may improve the effectiveness of therapy. To identify potential biomarkers, we downloaded microarray datasets of PD from the Gene Expression Omnibus database. Differentially expressed genes (DEGs) between PD and normal control (NC) groups were obtained, and the feature selection procedure and classification model were used to identify optimal diagnostic gene biomarkers for PD. A total of 1229 genes (640 up‐regulated and 589 down‐regulated) were obtained for PD, and nine DEGs (*PTGDS*,*GPX3*,*SLC25A20*,*CACNA1D*,*LRRN3*,*POLR1D*,*ARHGAP26*,*TNFSF14* and *VPS11*) were selected as optimal PD biomarkers with great diagnostic value. These nine DEGs were significantly enriched in regulation of circadian sleep/wake cycle, sleep and gonadotropin‐releasing hormone signaling pathway. Finally, we examined the expression of *GPX3*,*SLC25A20*,*LRRN3* and *POLR1D* in blood samples of patients with PD by qRT‐PCR. *GPX3*,*LRRN3* and *POLR1D* exhibited the same expression pattern as in our analysis. In conclusion, this study identified nine DEGs that may serve as potential biomarkers of PD.

**Abbreviations**
ARHGAP26: Rho GTPase activating protein 26
AUC: area under the ROC curve
CACNA1D: calcium voltage‐gated channel subunit alpha‐1D
DEG: differentially expressed gene
FDR: false discovery rate
GEO: Gene Expression Omnibus
GO: Gene Ontology
KEGG: Kyoto Encyclopedia of Genes and Genomes
MJD: Machado–Joseph disease
NC: normal control
PD: Parkinson's disease
PPI: protein–protein interaction
PTGDS: prostaglandin D2 synthase
qRT‐PCR: quantitative real‐time polymerase chain reaction
ROC: receiver operating characteristic
SVM: support vector machine
TNFSF14: TNF superfamily member 14
VGCC: voltage‐gated calcium channel

As a progressive motor neurodegenerative disorder, Parkinson's disease (PD) is the second most common neurodegenerative disorder after Alzheimer's disease among the elderly 1. PD, with the typical symptoms of resting tremor, bradykinesia, rigidity and postural instability, is defined primarily as a movement disorder and is pathologically characterized by degeneration of nigrostriatal dopaminergic neurons and the presence of Lewy bodies (misfolded α‐synuclein) in the surviving neurons 2. Although research into PD has been performed for nearly a century, there is still a large amount of work to be done exploring the pathogenesis of the disease. It is believed that the interaction of genetic factors, environmental factors and aging together contribute to the disease 3. Up to now, the current therapeutics for PD only alleviates the symptoms, and when the full‐blown syndrome occurs, there is no disease‐modifying way available to treat the disease. It is expected that applying prodromal premotor treatment to PD will slow down or stop the neurodegenerative process, leading to better quality of life of PD‐diagnosed patients. Accordingly, it is imperative to identify useful biomarkers for early diagnosis of PD, especially prior to the onset of motor symptoms 4.

With advances in various high‐throughput technologies, a number of key genes have been identified as diagnostic or prognostic biomarkers for various diseases, such as cancer and neurodegenerative disorders, by microarray or other high‐throughput technologies. Chen *et al*. 4 provided a set of novel miRNA candidates, including up‐regulated miR‐27a and down‐regulated let‐7a, miR‐142‐3p, let‐7f and miR‐222, for detecting PD. Chi *et al*. 5 proposed five significantly down‐regulated mRNAs and three significantly down‐regulated miRNAs to serve as useful clinical diagnostic markers. Compared with a single microarray study, integrated analysis of multiple microarrays could identify differentially expressed genes (DEGs) with more accuracy, and increase the statistical power.

In the present study, we identified DEGs by comparison between patients with PD and normal control (NC) groups by performing an integrated analysis of multiple microarray datasets. By using a feature selection procedure and classification model, the optimal diagnostic gene biomarkers for PD were obtained and their potential functions in PD were further analyzed by functional annotation. This study endeavored to better understand the molecular events and pathways of PD and represents an avenue for the exploration of new diagnostic strategies for PD.

## Materials and methods

### Microarray expression profiling in Gene Expression Omnibus

The Gene Expression Omnibus (GEO), developed and maintained by the National Center for Biotechnology Information (NCBI), is the largest database of high‐throughput gene expression data. The microarray datasets of PD were retrieved from the GEO database (http://www.ncbi.nlm.nih.gov/geo) by searching keywords (‘parkinson disease’ [MeSH Terms] OR Parkinson's disease [All Fields]) AND ‘Homo sapiens’ [porgn] AND ‘gse’ [Filter]. Datasets whose type was ‘Expression profiling by high throughput sequencing’ and which met the following criteria were included in our study: (a) the selected datasets were mRNA transcriptome data of the whole genome; (b) the data were derived from blood samples of patients with PD and NCs; (c) the datasets were standardized or raw datasets; (d) the sample size of the datasets was was ≥ 50. Two datasets (GSE99039 and GSE6613) were treated as training sets, and another dataset (GSE72267) served as a validated set. For demographic and clinical characteristics of individuals in these three datasets, refer to 6, 7, 8.

### Data preprocessing

The probes corresponding to multiple genes were removed. Among multiple probes corresponding to the same gene symbols, the probe with the largest average expression of the gene was retained for the following research.

### Identification of DEGs between PD and NCs

MetaMA, a meta‐analysis program for MicroArrays available in the r package, was used to identify the DEGs between PD and NC groups. The threshold was defined as *P*‐value < 0.05.

### Identification of optimal diagnostic gene biomarkers for PD

The lasso algorithm was used with the glmnet package (https://cran.r-project.org/web/packages/glmnet/) to reduce the dimensions of the data. We performed single 10‐fold cross‐validation cycles with the coordinate descent algorithm for each fold and found regularization parameters that led to the smallest average mean squared errors across all folds. The more optimal DEGs between PD and NCs were selected.

To further identify diagnostic value of the optimal genes, feature selection procedures were conducted as follows: (a) The importance value of each DEG was ranked according to the mean decrease in accuracy by using random forest analysis; (b) the optimal number of features was found by subsequently adding one DEG at a time in a top‐down forward‐wrapper approach; and (c) by using the support vector machine (SVM) at each increment, the accuracy was assessed and the optimal diagnostic gene biomarkers for PD were identified. Based on the obtained optimal diagnostic gene biomarkers for PD, we established the SVM model by using the e1071 package (https://cran.r-project.org/web/packages/e1071/index.html) in r; we established the random forest model by using the ‘random forests’ packet (https://cran.r-project.org/web/packages/randomForest/); we established the decision tree model by using the ‘rpart’ packet (https://cran.r-project.org/web/packages/rpart/index.html). The diagnostic ability of these three models was accessed by obtaining the area under a receiver operating characteristic (ROC) curve (AUC), accuracy, sensitivity and specificity.

### Functional annotation

To uncover the biological functions and detect the potential pathways of optimal diagnostic gene biomarkers for PD, the online software genecodis3 (http://genecodis.cnb.csic.es/analysis) was used to perform the functional annotation, including Gene Ontology (GO) classification (molecular functions, biological processes and cellular component) and Kyoto Encyclopedia of Genes and Genomes (KEGG) pathway enrichment. Statistical significance was defined as false discovery rate (FDR) < 0.05. The FDRs were calculated by using the Benjamin–Hochberg procedure.

### Protein–protein interaction network construction

The optimal diagnostic gene biomarkers for PD were scanned with the Biological General Repository for Interaction Datasets (BioGrid, http://www.uniprot.org/database/DB-0184). A protein–protein interaction (PPI) network was then constructed by using cytoscape software (version 3.5.0, http://www.cytoscape.org) in order to further explore the biological functions of the biomarkers.

### Confirmation of DEGs

Eight blood samples from five normal control people and three patients who were diagnosed as PD were collected. PD patients with a history of autoimmune diseases, anaphylaxis, immune deficiency, diabetes, heart disease, stroke, arteriosclerosis, psychosis, malignant tumor, severe cognitive impairment or central nervous system infection were excluded. Healthy controls matched by sex, age and education level were recruited in our study, and were excluded if there was nervous system disease, a history of mental illness or an adverse drug history. Written informed consents were obtained from all participants in this study. The research protocols complied with The Code of Ethics of the World Medical Association (Declaration of Helsinki) and the study was approved by the ethical committee of Dongying People's Hospital.

Total RNA was extracted using RNAliquid (Huitian, Beijing, China). By using FastQuant cDNA (Tiangen, Beijing, China), we generated cDNA from 1 μg extracted RNA. Quantitative PCR was performed with SuperReal PreMix Plus (SYBR Green) (Tiangen) in an ABI7300 real‐time PCR system (Applied Biosystems, Foster, CA, USA). By using the $2^{-\Delta \Delta C_{t}}$ method, relative gene expression was analyzed. Statistical significance was assessed by one‐way ANOVA. The expression levels of the selected genes were normalized against the *GAPDH*. The PCR primers used in this study are displayed in Table 1.

**Table 1:** The primers used in qRT‐PCR experiments

Gene	Primers (5′–3′)
*GAPDH*	Forward: 5′ GGAGCGAGATCCCTCCAAAAT 3′
	Reverse: 5′ GGCTGTTGTCATACTTCTCATGG 3′
*GPX3*	Forward: 5′ ACCCTCAAGTATGTCCGACCA 3′
	Reverse: 5′ GGTCAGATGTACCCAGGAGCT 3′
*SLC25A20*	Forward: 5′ GCAGTGATGATCCGAGCCTTC 3′
	Reverse: 5′ TCTCCTCAACGACAGCTTCCA 3′
*LRRN3*	Forward: 5′ TGGTACCATTGAGTCTCTGCCA 3′
	Reverse: 5′ TGCCGAACATTCTGACCTTGG 3′
*POLR1D*	Forward: 5′ CTGAAGGCGAGAGGAAGACAG 3′
	Reverse: 5′ GGTACCTCGAGTCTGAATGCG 3′

## Results

### Identification of DEGs between PD and NCs

In this study, two gene expression microarray datasets, namely GSE99039 and GSE6613, were enrolled for the integrated analysis (Table 2). A total of 1229 DEGs, including 640 up‐regulated genes and 589 down‐regulated genes, were detected in PD.

**Table 2:** List of mRNA study samples from GEO

GEO accession	Author	Platform	Samples (N : P)	Year	Country
GSE99039	Amar D	GPL570 [HG‐U133_Plus_2] Affymetrix Human Genome U133 Plus 2.0 Array	233 : 205	2017	Israel
GSE72267	Roncaglia P	GPL571 [HG‐U133A_2] Affymetrix Human Genome U133A 2.0 Array	19 : 40	2015	Italy
GSE6613	Scherzer CR	GPL96 [HG‐U133A] Affymetrix Human Genome U133A Array	22 : 50	2007	Denmark

### Optimal diagnostic gene biomarkers for PD

Based on reduced dimensions of the data, we obtained 25 DEGs between PD and NCs by using the lasso algorithm (Table 3).

**Table 3:** Differentially expressed mRNAs between PD and normal control after reduced dimensions of data. ES, effect size

ID	Symbol	Combined ES	*P*‐value	FDR	Regulation
5730	*PTGDS*	0.55350343	1.59E‐10	1.98E‐06	Up
54674	*LRRN3*	−0.447409685	3.60E‐07	0.000447953	Down
8740	*TNFSF14*	0.424933035	9.17E‐07	0.000759974	Up
51561	*IL23A*	−0.423333989	1.02E‐06	0.000789527	Down
23092	*ARHGAP26*	0.380421675	8.27E‐06	0.003114467	Up
2878	*GPX3*	0.412451433	3.14E‐05	0.006979377	Up
7056	*THBD*	0.390970585	3.22E‐05	0.006979377	Up
246126	*TXLNGY*	0.362737505	3.39E‐05	0.006979377	Up
6192	*RPS4Y1*	0.360917753	3.62E‐05	0.007060049	Up
51082	*POLR1D*	−0.339807664	6.01E‐05	0.009568719	Down
11328	*FKBP9*	0.445852125	0.000134479	0.015715772	Up
7280	*TUBB2A*	−0.324660659	0.000143781	0.016000973	Down
222658	*KCTD20*	0.32072981	0.000252398	0.02036717	Up
79960	*JADE1*	0.277092679	0.001245194	0.05016542	Up
79096	*C11orf49*	−0.262382026	0.002737984	0.079974141	Down
7145	*TNS1*	−0.246923009	0.004042499	0.102313913	Down
8435	*SOAT2*	−0.240853329	0.006551083	0.132374478	Down
776	*CACNA1D*	0.348515061	0.008196295	0.150106032	Up
55823	*VPS11*	−0.219797536	0.013025479	0.193159457	Down
788	*SLC25A20*	0.419278937	0.016440044	0.218264276	Up
745	*MYRF*	−0.313968366	0.018317776	0.229702324	Down
138151	*NACC2*	0.418849159	0.035769967	0.321790421	Up
1777	*DNASE2*	0.25753019	0.038446143	0.332709069	Up
1791	*DNTT*	−0.276654672	0.039502997	0.336086867	Down
5087	*PBX1*	−0.256786543	0.040926883	0.342953477	Down

**Figure 1:**
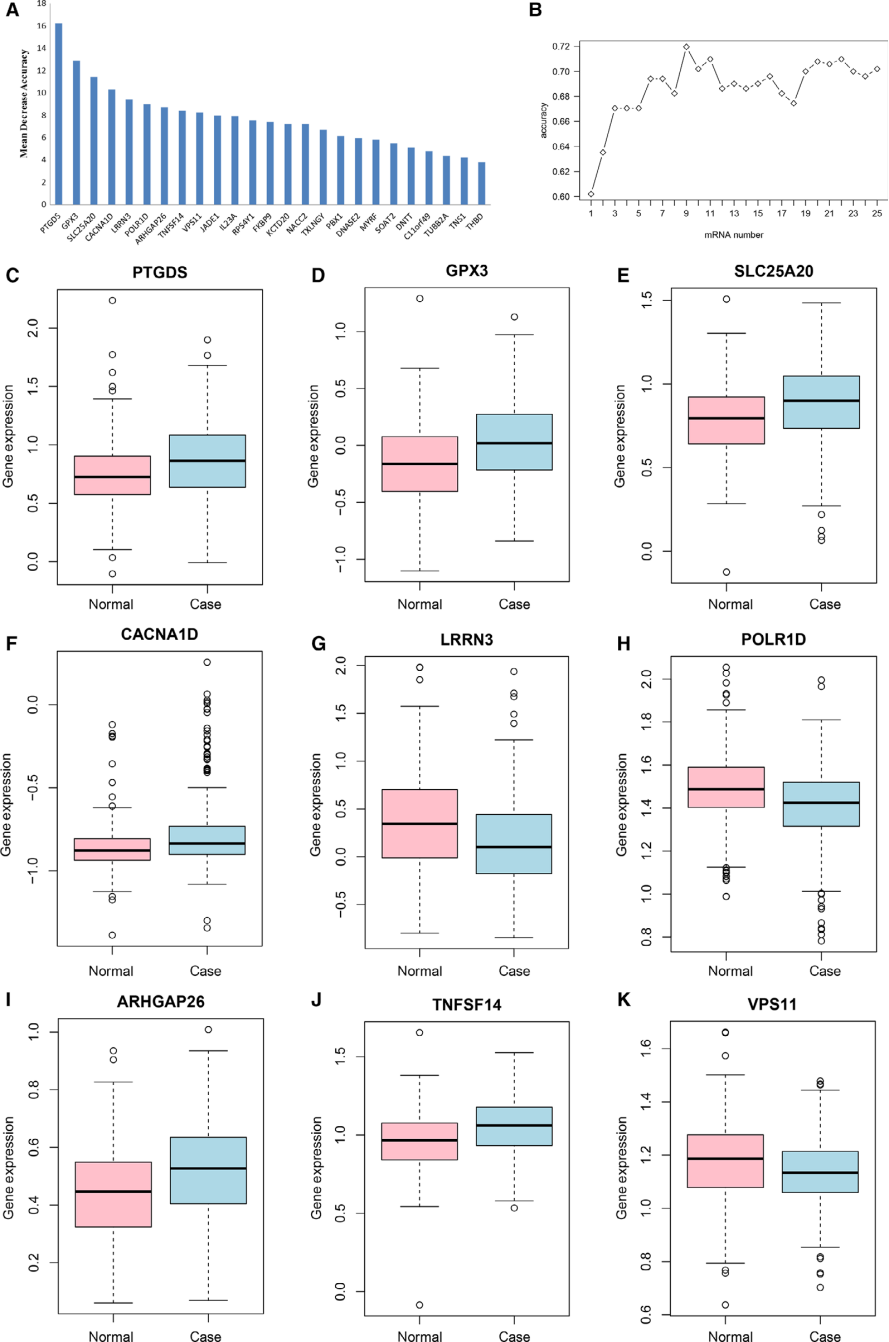
Identification of optimal gene biomarkers for PD. (A) The importance value of each DEG ranked according to the mean decrease in accuracy by using a random forest analysis. (B) The variance rate of classification performance when increasing numbers of the predictive DEGs. (C–K) Box‐plots displaying the expression levels of *PTGDS* (C), *GPX3* (D), *SLC25A20* (E), *CACNA1D* (F), *LRRN3* (G), *POLR1D* (H), *ARHGAP26* (I), *TNFSF14* (J), and *VPS11* (K); the *y*‐axis represents gene expression levels.

All these 25 DEGs were ranked according to the mean decrease in accuracy with the random forest analysis (Fig. 1A). Ten‐fold cross‐validation result indicated that the average accuracy rate of nine DEGs reached the highest point for the first time (Fig. 1B) and we defined these nine DEGs as the optimal diagnostic gene biomarkers for PD. Box‐plots displaying the expression levels of these nine DEGs between PD and NCs are shown in Fig. 1C–K.

**Figure 2:**
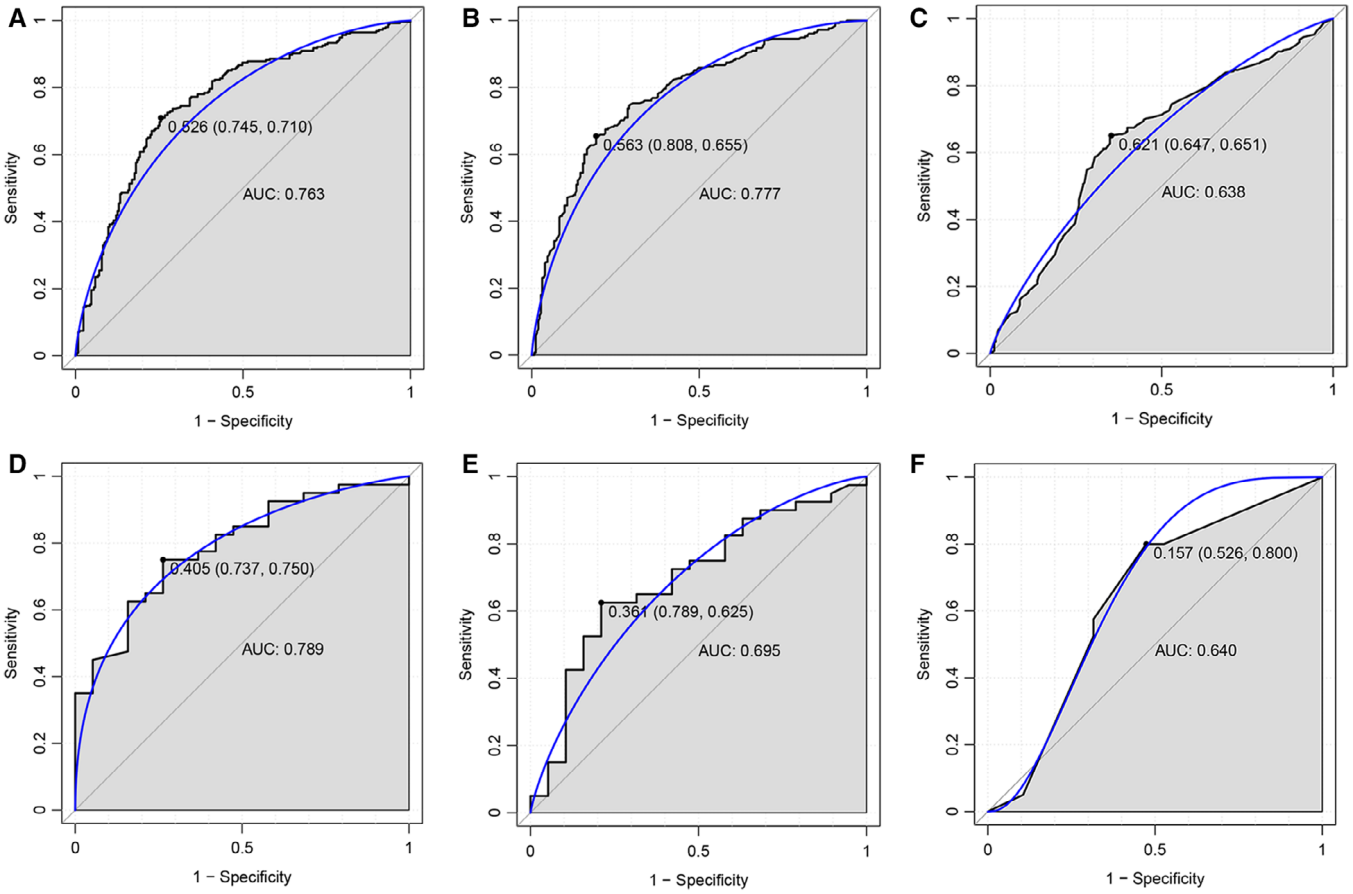
ROC results. (A–C) The ROC results of SVM (A), random forest (B) and decision tree (C) models based on the nine DEGs between PD and NCs. (D–F) The ROC results of SVM (D), random forest (E) and decision tree (F) models of the confirmation by dataset GSE72267 for these three models. The value before the parenthese represents the cut‐off. The values in the parenthese represent the specificity and sensitivity, respectively. The value below represents the AUC.

Based on these nine DEGs between PD and NCs, the SVM, random forest and decision tree models were established. The AUC of the SVM model was 0.763 and the sensitivity and specificity of the SVM model were 71.0% and 74.5%, respectively (Fig. 2A). The AUC of the random forest model was 0.777 and the sensitivity and specificity of the random forest model were 65.5% and 80.8%, respectively (Fig. 2B). The AUC of the decision tree model was 0.638 and the sensitivity and specificity of the decision tree model were 65.1% and 64.7%, respectively (Fig. 2C). GSE72267 (Table 2) was used to confirm these three models, and the ROC results are displayed in Fig. 2D–F.

### Functional annotation

Gene Ontology enrichment analysis revealed that these nine DEGs were significantly enriched in regulation of circadian sleep/wake cycle, sleep (FDR = 0.0114558), T cell activation (FDR = 0.0202354), extracellular space, extracellular region (FDR = 0.00210323), prostaglandin‐D synthase activity (FDR = 0.01216), tumor necrosis factor receptor binding (FDR = 0.015769) and voltage‐gated calcium channel activity (FDR = 0.0159844). According to a KEGG pathway enrichment analysis, several pathways were significantly enriched, including Alzheimer's disease (FDR = 0.0449926) and the calcium signaling pathway (FDR = 0.0452304).

### PPI network construction

**Figure 3:**
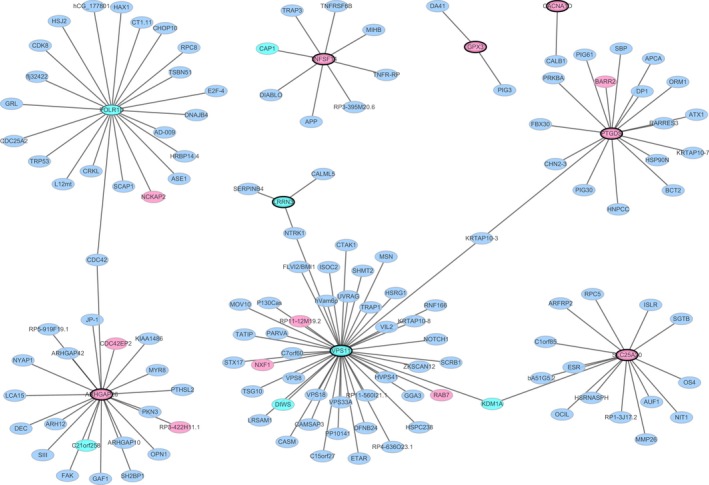
PPI network. The red and green ellipses represent proteins encoded by up‐ and down‐regulated differentially expressed mRNAs between PD and normal controls. Blue ellipses represent other proteins. Ellipses with a black border are the nine DEGs which were selected as the optimal diagnostic gene biomarkers for PD.

The PPI network contained 138 nodes and 133 edges. VPS11 (degree = 44), POLR1D (degree = 22) and ARHGAP26 (degree = 21) were the three hub proteins of the PPI network (Fig. 3).

### qRT‐PCR confirmation

**Figure 4:**
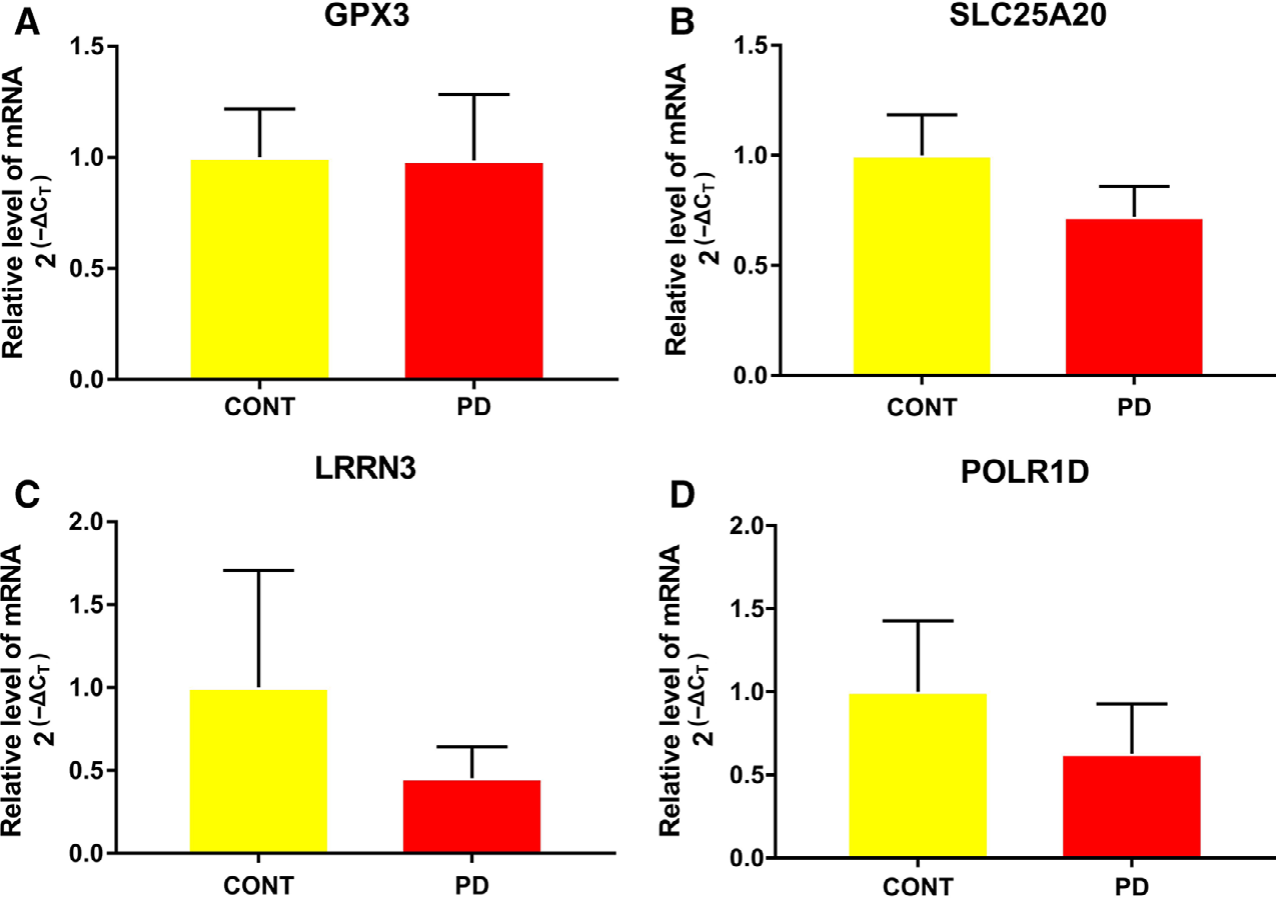
The qRT‐PCR results of DEGs between PD and NCs. (A) *GPX3*, (B) *SLC25A20*, (C) *LRRN3*, and (D) *POLR1D*. The *P*‐value > 0.05 was assessed by one‐way ANOVA. The error bars represent SD. *n* = 3.

To verify the expression of DEGs in our integrated analysis, the expression of four genes, namely *GPX3*,* SLC25A20*,* LRRN3* and *POLR1D*, was selected randomly for testing by quantitative real‐time polymerase chain reaction (qRT‐PCR). Based on our integrated analysis, *GPX3* was up‐regulated while *LRRN3* and *POLR1D* were down‐regulated in PD compared to NCs. Except for *SLC25A20* (which may be due to its relative large FDR value), expression trends of three genes (*GPX3*,* LRRN3* and *POLR1D*) in the qRT‐PCR results were consistent with that in our integrated analysis, generally (Fig. 4).

## Discussion

In recent years, a number of microarray studies of PD have been performed, mainly from the brain regions or blood. The most affected brain region in PD is the substantia nigra in the midbrain but the analysis of this region from postmortem PD brains may only highlight genes linked with changes in cellular composition 9. However, it is not readily possible to obtain postmortem brain tissue and this entails RNA quality concerns 10. This leads to the importance of studying blood samples in PD. It is easy to obtain the blood leukocytes, and the RNA can be obtained at high quality from them 11. Importantly, searching for blood‐based diagnostic markers is now an emerging field of study 12, 13. Hence, there is a growing interest in detecting blood biomarkers for PD. In this study, we performed an integrated analysis of PD microarray datasets to better reveal the pathogenesis of and develop novel diagnostic biomarkers for PD. A total of 1229 genes (640 up‐regulated and 589 down‐regulated) across the study were differentially expressed in PD with a *P*‐value < 0.05. We selected *GPX3*,* SLC25A20*,* LRRN3* and *POLR1D* to verify their expression level in PD. Except for *SLC25A20*, the other three genes displayed the same pattern in qRT‐PCR as that in the integrated analysis, which added evidence as to the reliability of the results in the integrated analysis.

Glutathione‐independent prostaglandin D synthase, encoded by prostaglandin D2 synthase (*PTGDS*), catalyzes the conversion of prostaglandin H2 to prostaglandin D2, a prostaglandin involved in pain, sleep and smooth muscle contraction/relaxation, and a potent inhibitor of platelet aggregation 14, 15. As is well known, sleep disorders are one of the symptoms of PD 16. A study has reported that additional PTGDS‐immunoreactive isoforms appear in many neurodegenerative disorders including Alzheimer's diseases and PD 17. In the integrated analysis, *PTGDS* was expressed more highly in PD compared with controls. In addition, in GO enriched analysis, *PTGDS* was enriched in regulation of the circadian sleep/wake cycle and in the sleep pathway. Importantly, up‐regulated *PTGDS* was identified as being a unique blood‐based signature capable of differentiating between patients with idiopathic Parkinson disease and controls in previous original studies of the microarrays 8. Hence, these results may suggest that increased *PTGDS* may play a crucial role in PD.

Duke *et al*. 18 reported that glutathione peroxidase 3 (*GPX3*) was more highly expressed in the medial nigral component of PD. In the integrated analysis, *GPX3* was found to be up‐regulated in PD as well. After GO and KEGG enriched analysis, *GPX3* was enriched in the pathways glutathione metabolic process, hydrogen peroxide catabolic process, response to oxidative stress, glutathione binding, and glutathione peroxidase activity, which are all associated with oxidative stress. Several studies in the literature have verified that oxidative stress plays an important role in the degeneration of dopaminergic neurons in PD 19, 20, 21, 22. Taken together, *GPX3* may be implicated in the development of PD.

Calcium voltage‐gated channel subunit alpha‐1D (*CACNA1D*) encodes a subunit which is one of the voltage‐gated calcium channels (VGCCs). VGCCs can be subdivided into various subfamilies, including the L‐type VGCCs (Ca_v_1 family), the T‐type VGCCs (Ca_v_3 family) and the P/Q‐type, R‐type and N‐type VGCCs (Ca_v_2 family) 23. Based on the study of Berger *et al*. 24, Ca_v_1.3 channels seem to be involved in neurodegenerative mechanisms associated with the development of PD, and a plethora of animal studies imply an important role of VGCCs in normal brain function and cognitive processes. Besides, the results of GO and KEGG enriched analysis showed that *CACNA1D* was enriched in pathways associated with calcium ion transport (e.g. regulation of calcium ion transport via voltage‐gated calcium channel activity, voltage‐gated calcium channel activity, calcium signaling pathway) and Alzheimer's disease, which is also a neurodegenerative disorder. Based on this evidence, the up‐regulation of *CACNA1D* detected in this analysis may be involved in PD.

The gene Rho GTPase activating protein 26 (*ARHGAP26*) was reported to be associated with neuropsychiatric diseases 25. In our study, *ARHGAP26* was significantly up‐regulated in PD when compared with controls, and it was also a hub gene in the PPI network, which further suggested that the gene *ARHGAP26* may be associated with PD.

To our best knowledge, there is no report to link TNF superfamily member 14 (*TNFSF14*) with PD until now. A previous study reported that *TNFSF14* shows an up‐regulation trend in Machado–Joseph disease (MJD) patients when compared with controls. MJD is a late‐onset polyglutamine neurodegenerative disorder caused by a mutation in the *ATXN3* gene, which encodes the ubiquitously expressed protein ataxin‐3 26. Similarly to PD, the non‐motor manifestations, such as sleep disorders, cognitive disturbances, psychiatric symptoms, olfactory dysfunction, peripheral neuropathy and dysautonomia, are also found in MJD 27. In the present study, *TNFSF14* also showed up‐regulation in PD compared with controls. Besides, according to GO and KEGG enrichment analysis, *TNFSF14* was enriched in the pathways associated with immunoregulation and apoptotic processes (for example, release of cytoplasmic sequestered nuclear factor‐κB, T cell proliferation, T cell activation, negative regulation of cysteine‐type endopeptidase activity involved in apoptotic processes). All signs indicated that increased TNFSF14 may play a vital role in PD.

In conclusion, nine DEGs were identified in this study that may serve as potential biomarkers of PD. Functional annotation of these DEGs in PD could contribute to exploring their precise roles in PD and further understanding the mechanism of PD at the molecular level. Among these nine DEGs, *PTGDS* was detected in GSE99039, and *SLC25A20* and *LRRN3* were detected in GSE72267. As Calligaris *et al*. 7 said, it is not surprising to find a very limited overlap in the identities of single genes between a study and those previously published, probably due to the complexity and heterogeneity of PD itself and differences in the cohort of patients and technical settings. Our study had a limitation. The sample size of the datasets used for the validation was small. Further validation studies with a larger sample size need to be performed to test the predictive power in diagnosis before clinical application.

## Conflict of interest

The authors declare no conflict of interest.

## Author contributions

FJ and QW designed the project. SS, GB and LG analyzed and interpreted the data. FJ and QW were major contributors in writing the manuscript. All authors read and approved the final manuscript.
